# Nutritional Factors in the Prevention of Atopic Dermatitis in Children

**DOI:** 10.3389/fped.2020.577413

**Published:** 2021-01-12

**Authors:** Thulja Trikamjee, Pasquale Comberiati, Enza D'Auria, Diego Peroni, Gian Vincenzo Zuccotti

**Affiliations:** ^1^Allergy and Immunology Unit, University of Cape Town Lung Institute, Cape Town, South Africa; ^2^Department of Clinical and Experimental Medicine, Section of Pediatrics, University of Pisa, Pisa, Italy; ^3^Department of Health Sciences, University of Milan, Milan, Italy; ^4^Department of Pediatrics, Vittore Buzzi Children's Hospital, University of Milan, Milan, Italy

**Keywords:** atopic dermatitis, breastfeeding, children, complementary foods, omega-3 long-chain polyunsaturated fatty acids, prevention, probiotics, vitamin D

## Abstract

Atopic dermatitis is one of the most frequent chronic skin diseases worldwide and often develops within the first few years of life. Recent advancements in our knowledge of its pathophysiology have brought to light the role of genetic predisposition and environmental triggers. With the increasing prevalence of allergic diseases, there is a strong need for a better understanding of the various modifiable eliciting factors of such conditions. The concomitant rise in food allergy and insights into the skin barrier function has highlighted the role of nutrition and diet in the prevention and modification of allergic disorders. Furthermore, the identification of the skin as an important route of sensitization, and the risk of progression to asthma later in life, stress the significance of optimizing our management of skin inflammation in the prevention of allergies. Many nutritional factors, including the type of maternal diet during pregnancy, the duration of breastfeeding, the epicutaneous exposure of allergenic food proteins in the first few years of life, the timing of the introduction of complementary foods, the supplementation of vitamins and probiotics/prebiotics during prenatal and early life, have been assessed as potential targets for the prevention of atopy and eczema. Here, we review the latest data addressing prenatal and perinatal nutritional and dietary interventions in the primary prevention of atopic dermatitis. Also, we define knowledge gaps and targets for future research in the prevention of atopic dermatitis.

## Introduction

Atopic dermatitis (AD) is a common inflammatory skin disease, which affects as many as one-fifth of all individuals (1) and is associated with a high financial and psychosocial burden for patients and their families (2, 3). The prevalence differs greatly in many parts of the world but has been found to have increased significantly in industrialized and developing countries in the last few decades (1, 4, 5). Changes in the exposome, due to urban expansion and socioeconomic growth, have led to greater energy consumption and waste production. The industrial revolution in the nineteenth century has led to increased exposure to air pollutants and chemical hazards, which has had an impact on the integrity of the physical epidermal barrier (6).

Recent findings in the pathogenesis of AD have revealed a complex interplay between impairment of the skin barrier function, environmental and nutritional factors, and immune dysregulation (6–9), which begins in early life. Some evidence suggests that AD is primarily a skin barrier defect (10, 11), which influences the development of sensitization and atopy (9, 12), and early AD may be a causative factor in developing food allergy (13). Indeed, the condition is often regarded as the first step of the “allergic march,” which leads to a progressive course of atopic illness, including food allergy, asthma, and allergic rhinitis.

As a result of the recent rise in allergic diseases worldwide, there has been a growing interest in the exploration of risk factors involved in the development of AD and epidermal barrier dysfunction (14, 15). Recent research has focused on the role of dietary and nutritional intervention in early life for the prevention of allergic diseases, as these factors are modifiable and can influence the immune system maturation in a crucial phase of its development (16).

**Figure 1:**
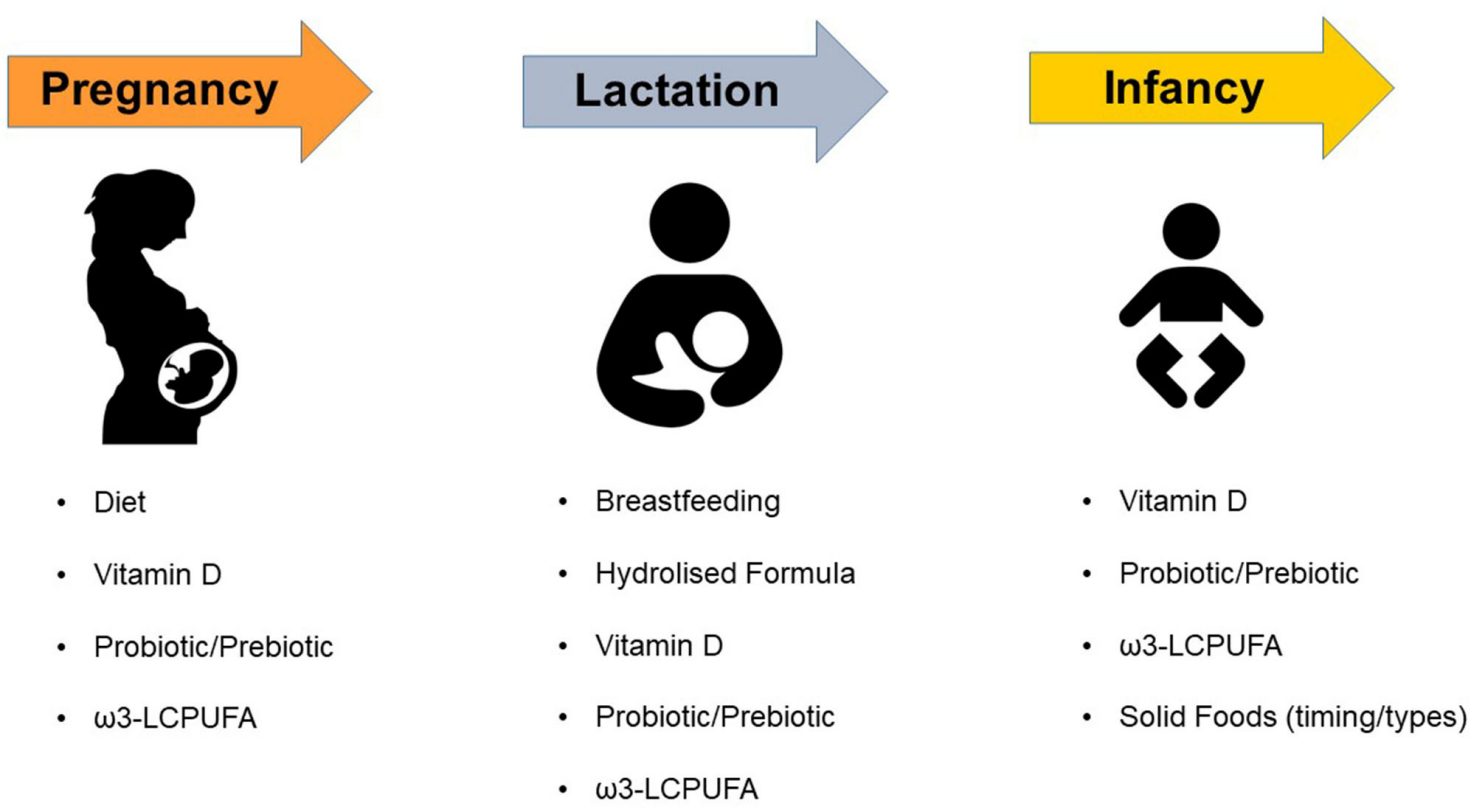
Dietary and Nutritional factors that may affect the risk of atopic dermatitis in children. ω3-LCPUFA, omega-3 long-chain polyunsaturated fatty acids.

In this review, we focus on currently available evidence on the nutritional and dietary factors that could be involved in the occurrence of AD and therefore could be targeted for the prevention of this disease (Figure 1).

## Maternal Diet During Pregnancy

Maternal prenatal nutrition and dietary diversity are crucial factors in a child's development. Some of the known health risks associated with intake at this time include obesity, hypertension, and diabetes (17). Several studies have assessed the role of the first 1,000 days after conception and their impact on the pathogenesis of allergic diseases (18). Current literature supports the hypothesis that the process of colonization by a healthy microbiome in the gut, airways, and skin in early life, can affect immune development and maturation, and the susceptibility to immune-mediated disorders later in life, including allergies (19, 20). The prenatal and early infancy period is a critical period for the type of microbiome colonization as well as for the maturation of immune responses, and exposure at this stage can promote immune tolerance (21, 22). The evidence for the prenatal maternal consumption of allergenic foods and their impact on allergic diseases is conflicting, and different for various foods. In addition, there have been many studies assessing the effect of prenatal nutritional exposures on early-life wheezing and asthma, and a paucity of data on AD. An earlier 2007 UK birth cohort found a beneficial effect on maternal oily fish consumption, with eczema at 5 years, but no association was found with other allergenic food groups investigated (23). This was consistent with a previous study by Dunstan et al. (24), which examined the effect of fish oil supplementation during pregnancy on early developing immune responses and outcomes in infants with atopic predisposition. A 2019 review of four longitudinal birth cohort studies found no significant effect of diverse Mediterranean diet patents in pregnancy on atopic outcomes in the offspring (25). Guidelines from Australasia, Germany, and the UK recommend eating fatty fish regularly during pregnancy (26). In 2015 Beckhaus et al. (27) showed that maternal consumption of various supplements (including vitamins C, D, E) had a protective effect on early life wheezing, but this did not extend to other atopic conditions. A recent cohort study found that the higher maternal intake of meat is associated with an increased risk of allergic rhinitis, wheezing, and AD in children (28). Overall, there is conflicting evidence on the effect of prenatal maternal consumption of certain food on the risk of allergy outcomes in childhood (29). Further studies are needed to assess the relationship between maternal dietary intake during pregnancy and long-term allergy outcomes in offspring. Furthermore, dietary diversity needs to be clearly defined to harmonize research into the effect of specific foods, considering geographic and cultural differences.

## Maternal Vitamin D Intake During Pregnancy

The worldwide rise in allergic diseases has paralleled a vitamin D (VD) deficiency epidemic in Westernized countries (30), which supports the hypothesis that VD might influence the development of allergies (30, 31). VD levels are mainly influenced by sun exposure but also by diet, which makes it an important modifiable factor in allergy prevention. It has been suggested that children born to mothers with low VD intake during pregnancy have an increased prevalence of AD (32). Cross-sectional studies also showed that children born during autumn and winter have a higher prevalence of AD compared with children born in spring and summer (33). In 2015 Beckhaus et al. (27) found that maternal intake of VD, vitamin E, and zinc during pregnancy was associated with a reduced risk of early life wheezing illnesses, but not of childhood-onset asthma or other atopic conditions in offspring. A recent meta-analysis from four prospective cohort studies showed that lower maternal VD serum level during pregnancy was associated with an increased risk of AD in offspring (34). Another recent meta-analysis of observational studies found no significant association between prenatal VD status (i.e., circulating 25-hydroxyvitamin D levels in maternal blood during pregnancy or cord blood at birth) and the risk of AD in offspring from age 1 to 9 years (35). However, a correlation between prenatal VD levels and the risk of AD was found at higher latitude, highlighting the effect of regional and geographic changes (35). More research is needed to analyze the influence of VD maternal status on the occurrence of AD in childhood.

## Breastfeeding

The is conflicting evidence on the relationship between breastfeeding and allergy risk, with some studies reporting protective effect against AD development, while others showing no effect or even an increased risk for AD occurrence (36). Still, international scientific societies recommend exclusive breastfeeding for at least 4–6 months for primary prevention of allergic disease (37, 38). Breastmilk supports diverse microbial colonization and drives the immune system maturation of the newborn (39–41). Breastfeeding has been associated with decreased morbidity and mortality in infants and lower incidence of allergic diseases (42). A 2005 birth cohort study enrolling 4,089 children showed that exclusive breastfeeding for ≥4 months reduced the risk for developing AD at 4 years of age, irrespective of the concomitant presence of either family history of atopy, allergic sensitization, or asthma (43). A systematic review of 18 prospective studies demonstrated that exclusive breastfeeding for the first 3 months after birth is associated with a lower incidence of AD in childhood, even in the presence of a family history of atopy (44). In contrast, a subsequent systematic review of prospective cohort studies comparing breastfeeding with conventional infant formula feeding or partial breast-feeding in developed countries, revealed that exclusive breast-feeding for at least 3 months was not significantly protective against the development of AD (45). Finally, a recent meta-analysis found that exclusive breastfeeding for 3–4 months was associated with a reduced risk of early life AD (<2 years of age), but the quality of evidence was low (46). In summary, the effect of breastfeeding on the risk of AD remains controversial (36), possibly due to different study populations and designs, and requires more randomized controlled trials (RCTs).

## Hydrolyzed Formula Feeding

Elemental cow's milk formula and hydrolyzed cow's milk or soy formulas are often prescribed to infants with the intention to prevent allergic diseases. However, their role in allergy risk reduction is still unclear (47). Partially (pHF) and extensively (eHF) hydrolyzed protein formulas have been widely investigated for their role in allergy and AD prevention. Two earlier RCTs reported no significant difference between pHF and eHF for the prevention of allergic diseases and AD in children (48, 49). This finding was in contrast to an earlier study by Oldaeus et al. (50), who found a lower incidence of AD in at-risk infants fed with a casein-based eHF, compared with those receiving a whey-based pHF or standard cow's milk formula (CMF). The GINI study, a prospective, randomized, double-blind trial, conducted among at-risk children, found a lower risk of AD at 3 and 6 years of life among those children who received a whey-based pHF or a casein-based eHF in their first 4 months, compared to those receiving CMF (51). Interestingly, this finding was exclusive to eczema as hydrolysate nutrition did not have a preventive effect on asthma or childhood wheezing (51). In the nationwide ELFE birth cohort study on infant feeding (comparing breast milk only, pHF with hypoallergenic label [pHF-HA] or without a hypoallergenic label [pHF-non-HA], and CMF), pHF-HA use was not associated with a lower risk of AD (52). The difference in these outcomes could be explained by the fact that the GINI trial was based only on whey-based pHF, whereas the ELFE cohort considered all types on pHF-HA formulas (51, 52). Finally, a recent Cochrane review found that nutrition with hydrolyzed formula, particularly pHF compared to CMF, in the early days of infancy, does not prevent atopic diseases among non-exclusively breastfed infants (47).

## Postnatal Vitamin D Intake

VD is a pleiotropic hormone and its insufficiency represents a growing global health concern. The VD receptor has been found in numerous immune and non-immune cells, including keratinocytes, and current evidence demonstrates that it modulates the expression of more than 200 genes (53–55). In recent years, the relationship between VD serum levels and the prevalence and severity of AD has been widely studied. Peroni et al. (56) showed that the serum levels of the circulating form of VD, the 25-hydroxyvitamin D, were inversely related to AD severity, although this finding was not confirmed in other studies (57, 58). In a Norwegian study, Byremo et al. (59) randomly selected 30 children from 4 to 13 years of age with severe AD to settle in a tropical zone for 4 weeks, and 26 children with AD to remain in Norway. A significant improvement was observed in disease activity as well as in the quality of life in the group who moved in a tropical zone after 4 weeks and 3 months (59). In a double-blind RCT, in which 60 AD patients aged ≥14 years were randomized to receive either 1,600 IU/day of VD or placebo, authors showed a significant improvement in the active group after 60 days, regardless of the initial severity of AD, which suggests that VD supplementation may improve AD (53). In contrast, Back et al. (60) showed that greater intake of VD during childhood correlated with an increased risk of AD at 6 years of age. VD supplementation in infancy has also been associated with a reduced risk of sensitization to house dust mites at age 18 months, which is an important trigger for the occurrence and severity of AD (61). Even though there are promising results regarding the role and therapeutic use of VD in AD, currently available data are conflicting. RCTs are needed to establish the optimal dose, desired levels, duration of treatment, and effect of VD supplementation in both prevention and treatment of AD.

## Probiotics and Prebiotics

It has been hypothesized that an imbalance in the intestinal microbiota composition and metabolic function due to dietary and lifestyle changes may be involved in the pathogenesis of atopic disease (22). The activation of the IL-4/IL-13 axis in AD promotes the skin barrier breakdown and is associated with changes in the gut microbiota (62). Several studies examining the role of oral administration of prebiotics and probiotics in atopy have shown that alterations in gut microbiota composition can precede the occurrence of AD (22, 62). In an earlier case-control study, individuals with AD had lower intestinal concentrations of *Bifidobacterium* compared to healthy controls (63). The *Bifidobacterium* levels were also inversely correlated with AD disease severity, suggesting that intestinal flora might play a role in AD onset and severity (63). The KOALA birth cohort revealed that the presence of *Clostridium difficile* was associated with a higher risk of developing AD and other allergic diseases, while a stronger association was found with *Escherichia coli*, which conferred an exponential risk to AD only (22). Prenatal and postnatal treatment with *Lactobacillus* and *Bifidobacterium* strains have been shown to reduce the risk of AD in infants (62). In a recent double-blind RCT, that included 50 children (aged 4–17 years), Navarro-López et al. (64) reported that a mixture of *Bifidobacterium* strains was effective in reducing AD severity as measured by the Scoring AD (SCORAD) index. A meta-analysis by Huang et al. (65) confirmed this result with improved SCORAD scores in 568 children treated with different strains. In a 2 year follow-up RCT including 132 at atopy risk infants, authors found that the cumulative incidence for AD was lower in the group fed with a formula that contained a mixture of prebiotic oligosaccharides (13.6%) compared to the placebo group (27.9%) (66). A recent Cochrane review, which evaluated the effect of oral prebiotics for the prevention of allergy in infants, reported a significant reduction in AD (67). In summary, supplementation with specific probiotic strains may modulate gut bacteria, which may influence skin inflammation, protect some children against AD development, and be considered a useful therapy in the future (68). However, the strain-specific effects of probiotics make it difficult to make recommendations (Table 1). Future studies comparing strains and adopting a common method of outcome measurement (such as SCORAD) will greatly improve our data and clinical recommendations.

**Table 1:** Randomized and non-randomized studies on the use of pre and probiotics for the prevention of atopic dermatitis in children.

**References**	**Study Type**	**Population (*N*)**	**Time of exposure**	**Interventions**	**Outcomes**
Penders et al. (22)	Prospective birth cohort (KOALA birth cohort)	957 infants at 1 month	Postnatal	Detection of gut microbiota composition in stool and total and specific IgE in venous blood	Associations between microbial composition and atopy at 2 years
Watanabe et al. (63)	Case-control	30 AD cases, and 68 controls	Postnatal	Detection of fecal microflora, fecal IgA concentrations, IgA on the skin surface	Differences in fecal microflora between patients with AD and healthy control subjects
Huang et al. (65)	Systematic review and meta-analysis of 13 RCTs	13 RCTs of children <18 years with confirmed AD	Postnatal	Scoring AD (SCORAD) assessment following probiotic administration	Effect of probiotics in the treatment of AD
Arslanoglu et al. (66)	RCT	134, high-risk infants	Postnatal	2 arms: 8 g/L scGOS/lcFOS) or placebo (8 g/L maltodextrin) during the first 6 mo of life	Allergic manifestations and infections during the first 2 years
Osborn and Sinn (67)	Systematic review of RCTs	4 RCTs, including 1428 infants	Postnatal	Prebiotic mixtures in low-risk and high-risk infants	Allergy outcomes in the first 2 years of life
Tam-lim et al. (68)	Systematic review and meta-analysis of RCTs	22 RCTs including 28 different strains	Postnatal	2 arms—strain mixes vs. placebo Mix1 (*Bifidobacterium animalis subsp. lactis* CECT 8145, *Bifidobacterium longum* CECT 7347, and *Lactobacillus casei* CECT 9104); *Lactobacillus casei* DN-114001; and Mix6 (*Bifidobacterium bifidum, Lactobacillus acidophilus, Lactobacillus case*i, and *Lactobacillus salivarius*)	Efficacy of probiotic strains compared to placebo, on pediatric atopic dermatitis

## Omega-3 Long-Chain Polyunsaturated Fatty Acids

Several birth-cohort studies have reported that increased omega-3 long-chain polyunsaturated fatty acids (LCPUFA) intake during pregnancy may reduce the risk of AD, asthma, and sensitization to house dust mite (69). The supplementation of LCPUFA, through the administration of fish oil during pregnancy and early life, has been proposed for the prevention of allergic sensitization and atopic diseases (70, 71). LCPUFA influence cell membrane structure and function, and potentially modulate inflammatory responses by increasing cell membrane docosahexaenoic acid (DHA; 22:6 ω-3) and eicosapentaenoic acid (EPA; 20:5 ω-3), thus competing with the synthesis of inflammatory arachidonic acid (AA, 20:4, ω-6). This results in a reduction in prostaglandin E synthesis and inhibition of cytokine and immunoglobulin E (IgE) production (72). While some studies showed that maternal fish oil supplementation during pregnancy is associated with a lower incidence of AD in offspring (73, 74), other authors reported no differences in the incidence of allergic diseases (75, 76). Best et al. (77) recently published the long-term follow-up of the DOMInO trial (78), where pregnant women were randomized to receive either fish oil capsules (900 mg of ω-3 LCPUFA) or vegetable oil capsules without ω-3 LCPUFA (control group) daily from the 2nd trimester of gestation until birth. The longitudinal analysis of 706 at-risk offspring from the DOMIno trial showed no significant difference in the progression of allergic diseases between the active and control groups assessed at 1, 3, and 6 years (77, 78). Conversely, a different RCT reported protective effects of prenatal supplementation with ω-3 LCPUFA on the risk of IgE-mediated AD at 1 year, and on follow up at 2 years (73, 74). Finally, a 2015 Cochrane review found that ω-3 LCPUFA supplementation in pregnant or breastfeeding mothers was associated with a reduction in AD in high-atopy risk children aged 12 to 36 months (but not at any other time point) but concluded that there was “limited evidence” to support supplementation with LCPUFA during pregnancy and/or lactation for the prevention of allergic diseases in children (79). In summary, despite the presence of RCTs suggesting protective effects, the data are still inconsistent, and long term follow-ups are crucial to determine whether prenatal and early postnatal ω-3 LCPUFA supplementation may be of benefit as a primary prevention strategy for AD.

## Timing of Complementary Feeding

Contrary to previous belief, the delayed introduction of solids in an infant's diet does not reduce the risk of allergic sensitization and atopic diseases (80–83). In a 2011 birth cohort of more than 18,000 newborns and 1,000 AD cases, Chuang et al. (84) found no evidence of a protective effect of delayed introduction of solid foods to infant's diet on the risk of AD at 18 months of age, while a longer duration of breastfeeding increased this risk. A recent case-control study conducted by the HYGIENE Study Group demonstrated that early introduction of solids was inversely related to the risk of AD. Children weaned at 4 months had a lower AD risk (OR = 0.41, 95% CI, 0.20–0.87) compared to those exclusively breastfed, and weaning started at 5 months of age revealed similar results (OR = 0.39, 95% CI, 0.18–0.83) (85, 86). Moreover, findings from the ISAAC Phase II Study found no evidence that prolonged exclusive breastfeeding protected against eczema (87).

The early introduction of fish has been associated with a reduced risk of allergic sensitization in some reports (88, 89), probably due to its high content of LCPUFA (70–72). However, not all the studies confirmed this protective role of fish introduction on the development of allergic diseases (89, 90). Despite the discrepancies in findings, observational studies find that delaying the introduction of solids increases the risk of AD. The difficulty in accepting early weaning to prevent AD is strongly linked with the emphasis given to the nutritional and health benefits of exclusive breastfeeding. Whilst more robust evidence is being sought to specify food types, quantities, and timing, recommendations should be aimed at gradually integrating a more diversified diet from 4 months of age, in addition to breastfeeding.

## Conclusion

Globally, robust recommendations on dietary intake during pregnancy for the prevention of allergic diseases are sparse. Some guidelines recommend eating fatty fish or taking LCPUFA supplements during pregnancy to reduce AD in the offspring. From our review of common dietary interventional strategies, there is conflicting evidence to support such recommendations. A most recent systematic review of 17 RCTs and 78 observational studies found no consistent evidence of a clear benefit of nutritional factors in the alteration of the risk of AD in children (91). Long-term follow-up studies are essential to determine the true benefit of prenatal and early life dietary and nutritional interventions as a primary prevention strategy for AD.

## Author Contributions

TT and PC made substantial contributions to the conception, design, and acquisition of data. TT and PC drafted the initial manuscript. ED'A, DP, and GZ critically reviewed the manuscript for important intellectual content. All authors approved the final version of the manuscript.

## Conflict of Interest

The authors declare that the research was conducted in the absence of any commercial or financial relationships that could be construed as a potential conflict of interest.

## Abbreviations

AD: Atopic dermatitis
eHF: Extensively hydrolyzed milk formulas
LCPUFA: Long-chain polyunsaturated fatty acids
pHF: Partially hydrolyzed milk formulas
RCT: Randomized controlled trial
SCORAD: Scoring Atopic Dermatitis
CMF: Standard cow's milk formula
VD: Vitamin D.
